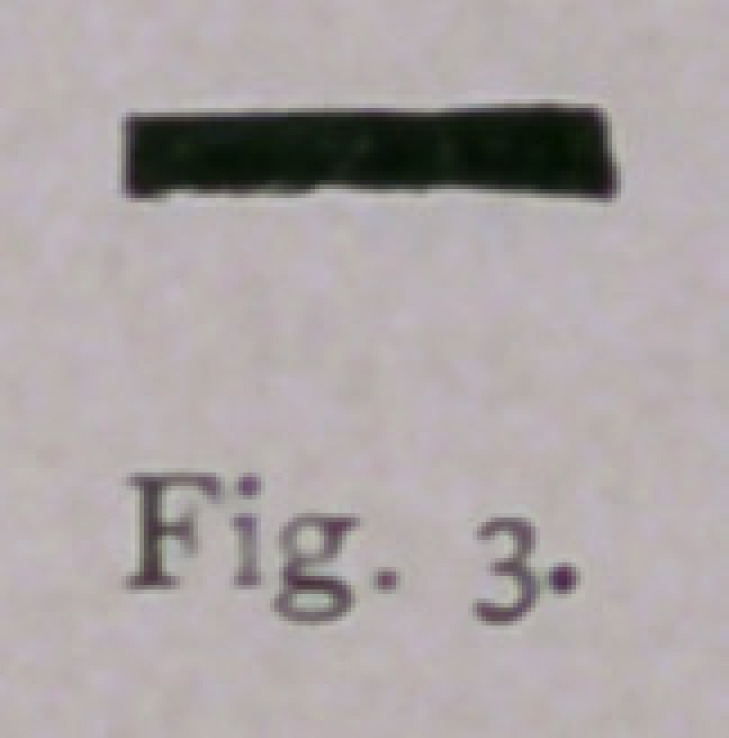# The Electro-Magnet in Removal of Steel from the Interior of the Eye*Read before the Western Branch of the New York State Medical Association, May 9, 1888.

**Published:** 1888-07

**Authors:** Alvin A. Hubbell

**Affiliations:** Professor of Diseases of the Eye, Ear and Throat, in the Medical Department of Niagara University, Buffalo, N. Y.; 212 Franklin Street


### *THE ELECTRO-MAGNET IN REMOVAL OF STEEL FROM THE INTERIOR OF THE EYE.* *

By ALVIN A. HUBBELL, M. D.,

Professor of Diseases of the Eye, Ear and Throat, in the Medical Department of Niagara University, Buffalo, N. Y.

One of the most serious accidents that can befall the eye is the introduction of a foreign body into its interior. In some cases vision is at once destroyed, while in nearly all, inflammatory action rapidly develops, and if any sight were previously left, it soon becomes lost by this process. Such injuries are, moreover, extremely liable to cause sympathetic disease of the other eye, and thus lead to total blindness. Exceptional cases have been recorded, in which the presence of a foreign body in the eye has been tolerated for weeks, months, and even years,

* Read before the Western Branch of the New York State Medical Association, May 9, 1888.

without suffering, and sometimes with more or less preservation of vision; but these are so rare as practically to have no weight against the rule that the foreign body must be removed or the eye will be lost.

The kinds of foreign bodies which are driven into the eye are various, according to the occupation and circumstances, and they may lodge at almost any point. They may also produce any extent of injury, from one scarcely discernible to one causing a total destruction of the eye. Under all circumstances, their removal is most desirable, and even imperative, so far as the preservation of vision is concerned; yet, to attempt this, involves one of the most uncertain and difficult procedures in surgery. All sorts of forceps, hooks, curettes, scoops, etc., have been devised for the purpose, and when the foreign body is situated in the anterior part of the eye, where it can be seen by the operator, some of them may be used with occasional success. But when it is lodged in the vitreous cavity or posterior part of the ball, the difficulties are increased many-fold, and it is often necessary to remove the whole eye in order to avert sympathetic disease of the other eye and save the patient from hopeless blindness. This is especially true when any substance except steel or iron enters the eye. When, however, it is the latter, the chances for removal and recovery are greatly increased by the use of the permanent or electro-magnet.

Every one knows that the magnet has the power of attracting iron and steel; and it is the practical application of this principle that enables the operator of the present time to save many eyes that, treated by the old methods, would be lost.

The permanent magnet was suggested for the removal of steel from the eye many years ago.* But the credit of starting the impetus which has led to its more general use is due to Dr. W. A. McKeown,† of Belfast, Ireland, who, in 1874, recorded several cases which he had successfully treated. Various forms of the permanent magnet have been devised, but one of the best is that

* It was suggested by Fabricius Hildanus, "*Opera Observationum et Curationum*," 1646; by Milhes, "*Observations of Medicine and Surgery*," 1745; by Morgagni, "*De Sedibus et Causis Morborum*," 1779; by Meyer, "*Mediz. Leitung vom Verein für Heilkunde in Preussen*," No. 2, 1842; by Himley, "*Die Krankheiten und Missbildungen des menschlichen Auges und deren Heilung*," 1843; by White-Cooper, *London* "*Lancet*," 1859; and by VonRothmund, in 1873, "*Centralblatt für Augenheilkunde*," 1880.

† "*British Medical Journal*," 1874.

of Dr. Greuning, of New York. This consists of several small cylinders joined together and armed at one extremity with a needle or point suitable for introduction into the eye.

The electro-magnet was first used by Hirschberg,* of Berlin, in 1875. His first instrument was of rude construction; but in 1877 a more perfect one was manufactured for him, which he fully described in 1881.

The electro-magnet, as used for extracting steel from the eye, consists essentially of a cylindrical core or nucleus of pure, soft iron, wound with several convolutions of insulated copper wire, which is connected with a galvanic cell or battery, and transmits the electric current for magnetizing the core. From one or both ends of the core are extended the points or needles to be introduced into the eye.

Several modifications of the electo-magnet have been presented to the profession, but the most important are those of Hirschberg, of Berlin; McHardy, of London; Snell, of Sheffield, and Bradford, of Boston. Hirschberg's seems to be the most bulky and clumsy, and McHardy's the lightest.

Fig. 1.

THE HUBBELL-PLUMB ELECTRO-MAGNET, ACTUAL SIZE. †

To this list I desire now to add another which had its origin at a time of emergency, and, as it appears to me, possesses sufficient merit to warrant me in calling the attention of the profession to it. It is shown in actual size in the accompanying cut, fig. 1.

---

* "Archives of Ophthalmology," Vol. X., 1881.

† For sale by Charles Pumb, Electrician, Buffalo, N. Y.

Its length, A to B, exclusive of the connecting-posts, is three and one-eighth inches (3.9 centimetres), and its diameter is a little less than three-fourths of an inch (17 millimetres). Its weight is three and one-fourth ounces. Its construction is somewhat different from that of other electro-magnets, inasmuch as the core is not solid iron, but consists of small, soft iron wires twisted together and around a larger central wire of sufficient size to receive the extensions or needles which are screwed into it at A. Furthermore, the coil is not one continuous wire, but consists of four wires, each running from the connecting-post, C, to the extremity, A, and back to the other connecting-post, D. This coil is supported at each end by the hard rubber caps, A and B, and is covered by a light, nickel-plated, brass jacket. The cap, A, is rounded, and the core at this extremity is tapped to receive the needles. These needles, of different sizes and lengths, are made of soft iron, and those shown in the cut are straight, but the smaller ones may be easily curved to suit any case. The extremity, B, receives the posts by which the magnet is connected with a battery, a quart, bichromate, single cell, being preferable. When the electric current from such a cell passes through the coil of this magnet, its attractive power is very great. The amount of iron which it suspends varies, however, with the length and size of the extension or needle used. The longer and smaller the latter, the less will it suspend. With the extension 7, fig. 1, it will raise and hold *thirty-one* ounces of iron; with one one-half of an inch long, measuring from the face of the instrument, and five thirty-seconds of an inch in diameter at its point, *twenty-eight* ounces; with one the same length, and point four thirty-seconds of an inch, *twenty-four* ounces; with one the same length, and point three thirty-seconds of an inch, *eighteen* ounces. I believe these measurements of power are far in excess of Bradford's magnet, which is often referred to as the strongest of the kind made. His magnet with extensions one-half inch long and five thirty-seconds, four thirty-seconds, and three thirty-seconds of an inch in diameter at their points, suspend, respectively, twenty, sixteen, and eleven ounces. This seems to be a great contrast of power in favor of the Hubbell-Plumb instrument.

The advantages claimed for this magnet are: its great power of attraction, its lightness, its small size, and its shape, most convenient for manipulation. These combined qualities make it a most valuable and desirable instrument, if not the best manufactured. Whatever merits, however, this magnet may possess, I cheerfully accord to its maker, Mr. Charles Plumb, of this city, a practical and scientific electrician. I simply suggested to him the principle upon which it should be constructed.

The diagnosis of steel in the eye is not always easy. In the majority of cases, the patient will state that something has struck his eye, but it will often be difficult to persuade him that anything has entered it Only the most careful examination with the ophthalmoscope, oblique illumination, etc., together with a consideration of the circumstances of the injury, will enable the surgeon to arrive at a satisfactory conclusion. Even then it is often impossible to make a correct diagnosis, on account of hemorrhage into the eye, mutilation of the parts, etc. In such cases, it has been suggested to apply the face or a large extension of the electro-magnet to the surface of the ball. If steel is in the eye, it is claimed that it will be attracted toward the magnet, and its movement will cause pain. While this is not altogether certain, it may be well to resort to this test in doubtful cases.

When it becomes probable or certain that steel is in the eye, the electro-magnet, for purposes of extraction, can be relied upon with much confidence. The manner of using it, and the kind of extension to be selected, whether straight or curved, long or short, large or small, depends upon the nature of the case and whether or not the steel has been seen and localized. In general, the needle of the magnet may be introduced through the original wound of the injury, or, if some time has elapsed since the accident, or for other reason, through an incision by the operator in nearer proximity to the steel. When a new opening is made, it is usually done through the sclerotic, between two of the recti muscles, avoiding, if possible, the ciliary body. Before making the incision, however, it is sometimes advisable to expose the sclerotic at this point, by making a conjunctival flap, or excising a piece of conjunctiva, and stopping all hemorrhage with hot water. Whether the magnet-needle be introduced through the injury-wound or the surgeon's incision, it should be cautiously directed toward the supposed locality of the steel, care being taken not to add further injury to the lens or other internal structures. If the steel is attracted to the magnet-needle, it often strikes the latter with a distinct click, which is both felt and heard, and is firmly held by it and withdrawn, unless the opening be too small, when it should be enlarged. After its removal, the conjunctival flap is replaced, or, if excised, the conjunctival wound is closed with fine sutures.

For this operation, the patient should be etherized. The magnet should also be previously tested to be certain that it is in good working order. The current of electricity should not be passed through it for too long a time, as it will, after a while, become heated. It can, however, be run for ten or fifteen minutes without being heated too much.

The results of treatment of steel in the eye with a good electro-magnet are gratifying in the extreme. It is not to be expected, however, that every eye will be saved by it, for the injury is frequently such that the eye is hopelessly lost from the beginning. But the success is so much greater than before it came into use, that the instrument marks an epoch in the therapeutics of this class of injuries.

The following cases have come under my observation:

Case I.—Michael Sullivan, aged forty-three, machinist. His left eye was struck with a piece of steel, June 18, 1884. He says the sight was destroyed at once. He was attended by another surgeon in this city, who enucleated the ball

On the morning of the 27th of the following August, two months after the accident to the left eye, while striking a "sett," a chip of steel flew into the right eye. It caused very little pain, the eye "watered" some, and vision was "pretty good." He at once sought advice, and was told that the steel could be seen in the back part of the eye. He entered St. Francis' hospital, and on the following day, the 28th, while anæsthetized, an unsuccessful attempt was made to remove the steel through the wound which the latter had made. I have been informed that an electro-magnet was used. The doctor, failing to extract the steel, and declining to make or allow any further efforts at its extraction, was discharged, and the patient was placed in my hands on the evening of August 30th, eighty hours after the accident.

The eye, at this time, was in constant pain, with a "pricking" sensation in its upper part, and vision was reduced to counting fingers at two feet. There was considerable lachrymation, the eyelids were somewhat swelled, the eyeball was very red, and the lower conjunctiva was chemotic. There was a wound at the junction of the lower part of the cornea and sclerotic two or three mm. long. This was still open with a bead of vitreous pressing through it. The pupil was not round, a piece of iris having probably protruded through the wound and been cut off. On using the ophthalmoscope, the fundus was found to be greatly obscured by either hemorrhage or inflammatory products, or by both. The steel could not be seen; I had the assurance, however, that it was in the vitreous, and it was evident that it had entered at the lower corneo-scleral junction, through the iris and suspensory ligament of the lens, and without wounding the lens, as this was perfectly transparent.

It was apparent that the steel must soon be extracted, or the eye would be lost and the patient blind. This, it seemed to me, might be done with the needle of a good electro-magnet introduced directly into the vitreous, through an incision of the sclerotic near the equator of the ball. The first thing needful, however, was an electro-magnet, and this I did not possess, and I did not know where I could at once obtain one. I, therefore, applied for aid to my friend, Mr. Charles Plumb, of this city. I told him what I wanted, and suggested the principle upon which an instrument should be made. He at once set his "genius" to work, and by ten A. M. the next day, he had one ready, of which the one above depicted is only a more finished copy.

At eleven A. M., August 31, just four days after the injury, I proceeded with my proposed operation. I was assisted by Drs. Tremaine, Lothrop, Mickle, and Montgomery, and by Mr. Plumb, who had charge of the magnet. After the patient was anæsthetized, I dissected a triangular flap of conjunctiva from the sclerotic at the external and lower part of the ball. The hemorrhage was checked with pieces of ice. I then made an incision with a cataract knife through the exposed sclerotic into the vitreous. This incision, about one sixth of an inch long, was directed antero-posteriorly between the external and inferior recti muscles and just in front of the equator of the eye. A few drops of yellowish fluid escaped through the incision. Mr. Plumb having the magnet in readiness, I introduced its needle, about half an inch long, into the vitreous cavity towards its center. Not feeling anything, I then directed its point toward the front of the eye, when I felt a click, which was distinctly heard both by myself and the bystanders. On withdrawing the needle, the steel came with it, firmly held in its magnetic grasp. It was a thin scale, and is shown in actual size in Fig. 2. The conjunctival flap was replaced and stitched at its apex, when it nicely covered and protected the sclerotic wound.

Fig. 2.

Very little reaction followed the operation, and both the original and new wounds healed kindly. Improvement began to take place immediately. After several months, the opacities of the vitreous had cleared away, and the patient was able to read the finest print slowly. The field of vision was left somewhat contracted, and the ophthalmoscope showed some marked choroidal changes. But the vision is still serviceable. The patient reads a great deal, and is enabled to earn an independent livelihood.

CASE II.—April 5, 1888, I was invited by Dr. F. W. Abbott, of this city, to examine an interesting case, which he has kindly permitted me to refer to in this paper. On April 3, 1888, Miss

Argus, about twenty years of age, was stitching leather with a sewing-machine, when the needle broke and a piece struck her right eye. She applied to Dr. Abbott for advice, not, however, suffering much distress or pain. On examination, there was scarcely any evidence of injury, except the anterior chamber was obscured with blood. A solution of atropia was ordered instilled, and she was asked to return in two days. At this time, I was invited to see the case. The blood had become absorbed, and the pupil was nearly at its maximum dilatation, thus permitting an easy examination of the fundus. The opthalmoscope showed all the meadia to be transparent, and a piece of the sewing-machine needle could be distinctly seen in the vitreous, projecting from the anterior and inner part of the cavity directly toward its center. A slight mark could be distinguished externally at the inner corneo-scleral junction, indicating the point of its entrance. It had evidently passed through the iris, and nearly through the ciliary body, by which it was now firmly held. The eye was slightly red and irritable.

Fig. 3.

Arrangements were made for an operation on April 6th, at which I assisted with my magnet. The patient being anæsthetized, the doctor excised a piece of conjunctiva immediately posterior to the point of entrance of the needle and over the inner ciliary region. The hemorrhage was stopped, first using pieces of ice, but afterwards cotton steeped in hot water, which was much more effective. He then made an atero-posterior incision directly through the sclerotic and ciliary body into the vitrous cavity, about one-sixth of an inch long. The magnet being in readiness, I introduced a small extension through the incision, and it at once grasped the broken needle, but without any click, and without loosening it. I held it steadily with the magnet while the doctor enlarged the incision anteriorly until it was entirely disentangled, when it was easily withdrawn, adhering to the magnet. Fig. 3 shows the size of the piece of needle extracted.

The conjunctival wound was closed by two sutures passed perpendicularly to the sclerotic incision. The patient was kept quietly in bed a few days, with the eye bandaged, and atropia was occasionally instilled.

I examined the eye four weeks after the operation, and it appeared to have fully recovered, and vision was, undoubtedly, as good as ever.

These two interesting cases, as well as numerous others in the practice of other surgeons, show the usefulness of the electro-magnet. Both eyes, without doubt, would have been lost, and one patient, at least, would have been blind, had it not been for this instrument.

212 FRANKLIN STREET.

## Figures and Tables

**Fig. 1. f1:**
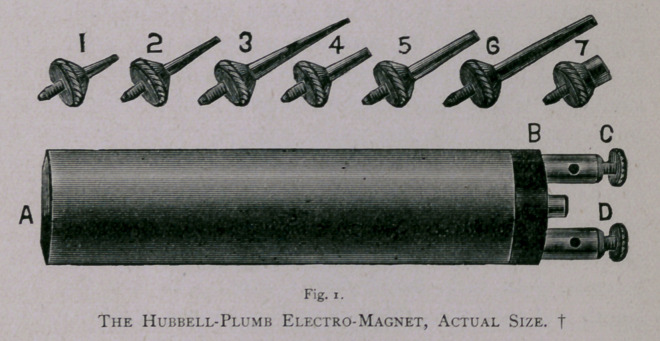


**Fig. 2. f2:**
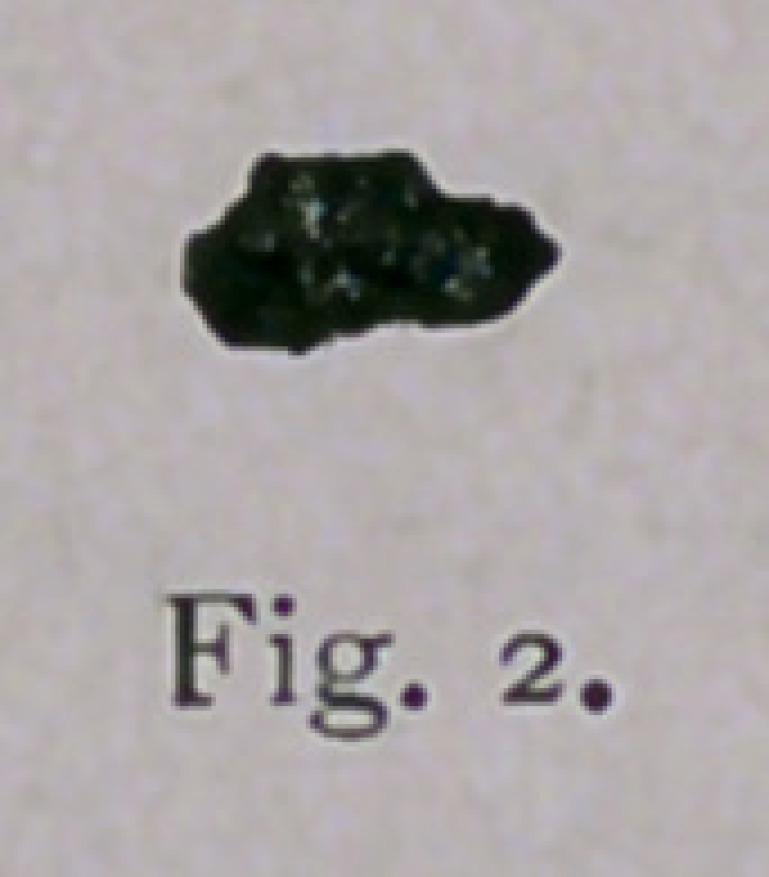


**Fig. 3. f3:**